# Transforming growth factor-β signalling controls human breast cancer metastasis in a zebrafish xenograft model

**DOI:** 10.1186/bcr3573

**Published:** 2013-11-07

**Authors:** Yvette Drabsch, Shuning He, Long Zhang, B Ewa Snaar-Jagalska, Peter ten Dijke

**Affiliations:** 1 Department of Molecular Cell Biology, Cancer Genomics Centre Netherlands, Leiden University Medical Center, Postbus 9600 2300, RC, Leiden, The Netherlands; 2 Centre for Biomedical Genetics, Leiden University Medical Center, Postbus 9600 2300, RC, Leiden, The Netherlands; 3 Institute of Biology, Leiden University, Einsteinweg 55, 2333, CC Leiden, The Netherlands; 4 Ludwig Institute for Cancer Research, Science for Life Laboratory, Uppsala University, Box 595, 75124 Uppsala, Sweden; 5 Current address: Life Sciences Institute, Zhejiang University, Hangzhou, Zhejiang 310058, China

## Abstract

**Introduction:**

The transforming growth factor beta (TGF-β) signalling pathway is known to control human breast cancer invasion and metastasis. We demonstrate that the zebrafish xenograft assay is a robust and dependable animal model for examining the role of pharmacological modulators and genetic perturbation of TGF-β signalling in human breast tumour cells.

**Methods:**

We injected cancer cells into the embryonic circulation (duct of cuvier) and examined their invasion and metastasis into the avascular collagenous tail. Various aspects of the TGF-β signalling pathway were blocked by chemical inhibition, small interfering RNA (siRNA), or small hairpin RNA (shRNA). Analysis was conducted using fluorescent microscopy.

**Results:**

Breast cancer cells with different levels of malignancy, according to *in vitro* and *in vivo* mouse studies, demonstrated invasive and metastatic properties within the embryonic zebrafish model that nicely correlated with their differential tumourigenicity in mouse models. Interestingly, MCF10A M2 and M4 cells invaded into the caudal hematopoietic tissue and were visible as a cluster of cells, whereas MDA MB 231 cells invaded into the tail fin and were visible as individual cells. Pharmacological inhibition with TGF-β receptor kinase inhibitors or tumour specific Smad4 knockdown disturbed invasion and metastasis in the zebrafish xenograft model and closely mimicked the results we obtained with these cells in a mouse metastasis model. Inhibition of matrix metallo proteinases, which are induced by TGF-β in breast cancer cells, blocked invasion and metastasis of breast cancer cells.

**Conclusions:**

The zebrafish-embryonic breast cancer xenograft model is applicable for the mechanistic understanding, screening and development of anti-TGF-β drugs for the treatment of metastatic breast cancer in a timely and cost-effective manner.

## Introduction

Transforming growth factor-β (TGF-β) system signals via serine/theronine kinase receptors and intracellular Smad transcriptional mediators to regulate a large number of biological processes [1]. Alterations of the TGF-β signalling pathway are implicated in many human diseases, including cancer (reviewed in [2]). Prior to tumour initiation and during the early stages of cancer, TGF-β often acts as a tumour suppressor; however at later stages it functions as a tumour promoter. As tumours develop they switch their response to TGF-β and utilise this factor as a potent promoter of cell motility, invasion, metastasis, and tumour stem cell maintenance (reviewed in [3,4]). Multiple signal transduction pathways, involving a range of signalling molecules, determine the effects of TGF-β influence on multiple aspects of tumour growth and progression. Further research on how this cytokine is capable of being a tumour suppressor turned into a tumour promoter is important for the development and informed use of potentially powerful TGF-β targeted therapies [5].

Over the past decade, zebrafish (*Danio rerio*) has become an important animal model for cancer, immune and stem cell research [6-8]. Zebrafish are ideal for identifying clinically relevant genes and compounds that regulate tumour progression. There is conservation between zebrafish and mammals of many molecular and cellular components that operate during tumourigenesis. In addition, the physiological responses in zebrafish embryo to a wide range of pharmacologically active compounds are comparable to those in mammalian systems [9]. High resolution *in vivo* analysis of tumour progression and the interactions between tumour cells and the host microenvironment [10,11] can be readily performed due to the transparency of zebrafish, in combination with the availability of various tissue-specific fluorescent reporter transgenic lines [12,13]. Several tumour transplantation assays with human and mammalian cells to study different aspects of tumour malignancies in embryo and adult zebrafish, such as tumour cell migration, proliferation, angiogenesis and tumour cell extravasation [6,12,14-16] have been developed. Many of these assays are simplistic and are limited to one selected step of tumour development, and thus, do not represent the full complexity of tumourigenesis in one model. A rapid and reproducible zebrafish embryonic xenograft model for simultaneous formation of a localized tumour and experimental micrometastasis, by intravascular injection of tumour cells into the blood circulation of zebrafish embryos, has been recently described by the group of Snaar-Jagalska [17]. They have shown that with non-invasive high-resolution imaging, the critical steps of tumour progression, including tumour vascularisation and tissue invasion, can be characterized.

We applied this xenograft model and focused our studies on the effect of misregulation of TGF-β signalling components in breast cancer invasion and metastasis. We have used breast cancer cell lines of which, in previous studies, we and others have shown that the invasive and metastatic behaviour in spheroid invasion and mouse xenograft models is dependent on TGF-β [18]. We demonstrated that the invasive and metastatic behaviour, corresponding with the cell grade of malignancy can be recapitulated within the zebrafish. Moreover, the effects obtained after inhibiting with TGF-β receptor and Smad function in fish mimicked the effects observed in mice. Importantly, an effector role for matrix metalloproteinases (MMPs) in invasion and metastasis was demonstrated in this model. The differences in invasive properties upon dysregulation of TGF-β signalling components and its effectors are seen with clarity unprecedented in other animal models, making it applicable in a pipeline for new drugs discovery.

## Material and methods

### Reagents and cell culture

Human cell lines (293 T, 3 T3, and MDA-MB-231) were maintained cultured at 37°C in DMEM-high glucose containing L-glutamine, 10% FCS and 1:100 Penicillin/Streptomycin (Pen/Strep) (Gibco, Invitrogen, Blijswijk, Netherlands). The MCF10A-derived breast epithelial cell lines M1, M2, and M4 (MCF10A or M1, MCF10AT1k.cl2 or M2, and MCF10CA1a.cl1 or M4) were maintained as previously described [19]. Zebrafish cell lines, ZF4 and PAC2 were maintained according to the American Type Culture Collection (Manassas, VA, USA) recommendations. Briefly, zebrafish cells were cultured at 28°C in DMEM containing L-glutamine, 10% FCS and 1:100 Pen/Strep (Gibco, Invitrogen, Blijswijk, Netherlands). The 293 T, 3 T3, MDA-MB-231, ZF4 and PAC2 cells were originally obtained from American Type Culture Collection. MCF10CA1a.cl1 (M4) cells were kindly provided by Dr Fred Miller (Barbara Ann Karmanos Cancer Institute, Detroit, MI, USA).

### Luciferase experiments

Cells were plated on day 0 in 24-well plates and transfected with constant total concentration of DNA in a given experiment on day 1. Cells were transfected with a TGFβ/Smad3-responsive promoter, firefly luciferase transcriptional reporter construct, CAGA_12_-luc [20], in conjunction with a β-galactosidase control for transfection efficiency. Cells were also transfected with zTGF-β1, reverse zTGF-β1, or an empty vector control. The same day, cells were either untreated or treated with TGF-β 1 ng/mL for 24 h. Cell lysates were harvested on day 2 and luciferase activity was measured. Experiments were performed in triplicate and repeated twice. The CAGA_12_ promoter-driven luciferase activity was normalized to the β-galactosidase activity, averaged, and plotted. Luciferase results were analysed by one-way analysis of variance (ANOVA). Representative experiments are shown.

### Fluorescent cell labelling

Non-fluorescent cells were labelled with the fluorescent cell tracker CM-DiI (Invitrogen, Blijswijk, Netherlands) according to the manufacturer’s instructions. Briefly, cells were grown to confluence in a T25-cm^2^ dish, and trypsinized. Subsequently, cells were washed with PBS, transferred to 1.5 ml Eppendorf tubes and centrifuged 5 min, at 1200 rpm. Cells were re-suspended in PBS containing CM-Dil (4 ng/ul final concentration). Cells stained with CM-Dil were incubated for 4 minutes at 37°C and then 15 minutes at 4°C. After this period cells were centrifuged for 5 minutes at 1,200 rpm, the supernatant discarded and cells re-suspended in media, centrifuged again and washed two times with PBS. Cells were suspended in DMEM or PBS for injection into the embryos.

### Lentiviral transduction

Lentivirus was produced by co-transfecting an mCherry PLKO plasmid and helper plasmids pCMV-VSVG, pMDLg-RRE (gag/pol), and pRSV-REV into HEK293T cells. Cell supernatants were harvested 48 h after transfection and used to infect cells or stored at -80°C. For stable cell lines, cells were infected at 20% confluence for 24 h with lentiviral supernatants diluted 1:1 with normal culture medium in the presence of 5 ng/mL polybrene (Sigma, Zwijndrecht, Netherlands). At 24 h after infection, cells were placed under puromycin (1 μg/ml) selection for one week, or collected for injection at 1 day after infection.

### Zebrafish maintenance

The Institutional Committee for Animal Welfare of the Leiden University Medical Center (LUMC) approved this study. Zebrafish and embryos were raised, staged and maintained according to standard procedures. The transgenic line Tg(fli1:GFP) was used in this study [11,12].

### Embryo preparation and tumour cell implantation

Dechorionized 2 days-post fertilisation (dpf) zebrafish embryos were anaesthetized with 0.003% tricaine (Sigma) and positioned on a 10-cm Petridish coated with 3% agarose. Single cell suspensions of fluorescent mammalian cells were re-suspended in PBS, kept at room temperature before implantation and implanted within 3 h. The cell suspension was loaded into borosilicate glass capillary needles (1 mm O.D. × 0.78 mm I.D.; Harvard Apparatus) and the injections were performed using a Pneumatic Picopump and a manipulator (WPI, Stevenage, UK). Approximately 400 cells (manually counted) were injected at approximately 60 μm above the ventral end of the duct of Cuvier (DoC), where the DoC opens into the heart. After implantation with mammalian cells, zebrafish embryos (including non-implanted controls) were maintained at 33°C, to compromise between the optimal temperature requirements for fish and mammalian cells [21]. For each cell line or condition, data are representative of at least three independent experiments with at least fifty embryos per group. Experiments were discarded when the survival rate of the control group was less than 80%.

### *In vivo* toxicity test of chemical compounds

Chemical compounds were added to the eggwater at 2 dpf for toxicity tests, or 24 h post implantation (hpi) for treatment, and refreshed every second day. Chemical compounds were: LY-294002 (Cell Signaling, Leiden, Netherlands), SB-431542 (Sigma, Zwijndrecht, Netherlands) and Gm6001 (Calbiochem, Amsterdam, Netherlands). For toxicity tests, embryo survival or malformation was scored daily. For treatment, after 5 days embryos were fixed overnight in 4% buffered paraformaldehyde (PFA) at 4°C. Embryos were placed in a glass-bottom 96-well plate (Greiner Bio One GmbH, Frickenhausen, Germany), and imaged as described.

### Microscopy and analysis

Fixed embryos were imaged in PBS-Tween. Fluorescent image acquisition was performed using a Leica MZ16FA stereo microscope, or a Leica SP5 STED confocal microscope. Confocal stacks were processed for maximum intensity projections with Leica software or Adobe Photoshop CS4 software. Images were adjusted for brightness and contrast using Adobe Photoshop CS4. Overlays were created using Adobe Photoshop CS4.

### Immunohistochemistry

Whole-mount immunohistochemistry of zebrafish was carried out as described [22]. Primary anti-phospho-Smad2 (p-Smad2) antibodies (Millipore, Amsterda, Netherlands) (1:200 dilution) and secondary antibodies (Alexa 647 anti-mouse; Invitrogen, Blijswijk, Netherlands) (1:200 dilution) were used for detection. Immunohistochemistry of zebrafish cell lines required cells to be plated in 12-well plates on coverslips. The methods for immunohistochemical staining of p-Smad2 are described in the manufacturer’s instructions. In brief, the slides were incubated with p-Smad2 antibody (1:2,000) overnight at 4°C, washed with PBS, and incubated with the secondary antibody (Alexa 488).

## Results

### Invasion and micrometastasis formation of human breast cancer cell lines in zebrafish

**Figure 1:**
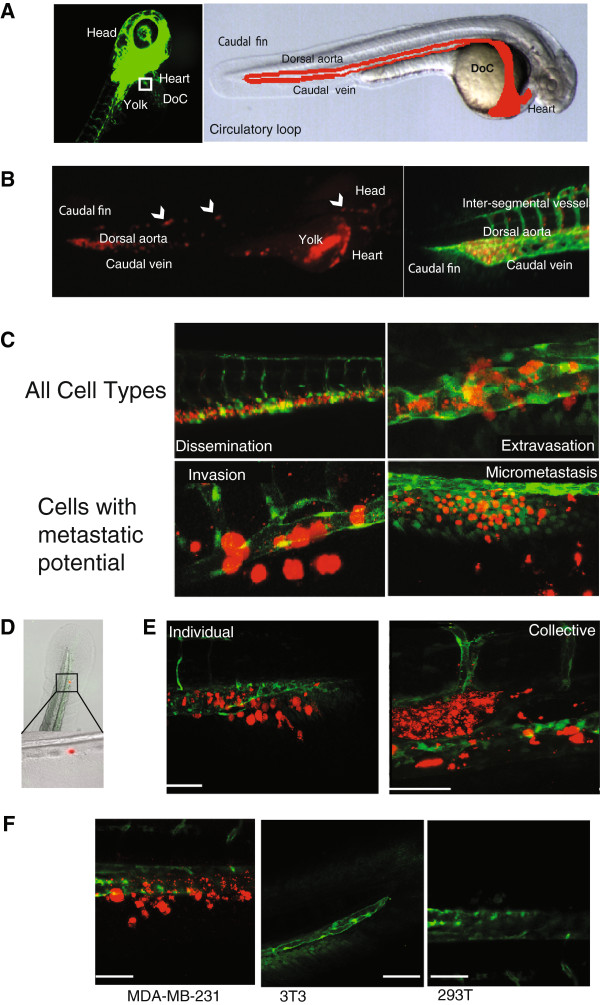
**Schematic of embryonic zebrafish and injections into the ducts of Cuvier. (A)** Fluorescent microscopy image of a 48 h-post fertilization (hpf) Tg(fli1:GFP) zebrafish. The boxed area represents the ideal injection site. The cells will then enter into the circulatory loop, as shown in the light microscopy image. The dorsal aorta, caudal vein, and ducts of Cuvier (DoC) are emphasized with red. **(B)** mCherry-labelled MDA-MB-231 cells injected into the DoC immediately disseminate throughout the vasculature. Image was taken 2 h-post implantation (hpi). A magnification of the tail region, displaying the vasculature (green), and cells (red) is shown. **(C)** Typical images of the various stages of cell survival in the zebrafish embryo. Only cells with metastatic potential are capable of undergoing invasion and micrometastasis. MDA-MB-231 cells (red) are shown. **(D)** A light microscopy image of the tail fin after injection with fluorescent polystyrene beads. Beads become lodged within the end of the circulatory loop. **(E)** MDA-MB-231 cells (red) displayed invasion as singular cells into the collagen fibres of the tail fin (scale bar = 100 μm). M2 and M4 (shown) displayed an invasive phenotype in with cells collectively cluster together (scale bar = 50 μm). **(F)** Only tumourigenic cells were capable of displaying invasive properties in the fish. MDA-MB-231 cells were compared to the motile, but non-invasive 3 T3 cells, and the weakly tumorigenic 293 T cells. Scale bar = 100 μm. Data are representative of three independent experiments with at least 50 embryos per group.

Human cancer cell lines have provided a rich source of propagatable material for the molecular and cellular characterization of cancer pathogenesis. The MCF-10A series of cell lines (MCF10A or M1, MCF10AT1k.cl2 or M2, and MCF10CA1a.cl1 or M4) represents the spectrum of progression from relatively normal breast epithelial cells (M1), pre-malignant (M2) to high-grade carcinoma capable of metastasis (M4) [19]. The MDA-MB-231 cell line is used extensively for the study of hormone-independent breast cancer. It is capable of forming tumours in immune-deficient mice, and has a high metastatic potential, thereby providing xenograft models for study cancer development *in vivo*[23]. These cell lines were fluorescently labelled, either with mCherry stable transfection or CmDiI, and were transplanted into the DoC of 2-day-old zebrafish embryos (Figure 1A) to study invasive and metastatic behaviour *in vivo*. Injection of invasive or metastastic cells directly into the blood circulation allows the cells to be distributed throughout the organism with the blood flow. This assay models the later stages in successful metastasis, without the formation of a primary tumour site [17]. Immediately after injection, the implanted cells hematogenously disseminate in the embryo (Figure 1B). Within the first 3 h, the tumour cells arrest within the dorsal aorta and caudal vein, and also some cells may penetrate into the smaller optic veins and the inter-segmental vessels (Figure 1B). The breast cancer cells M1 (low metastatic potential), M2 (moderate metastatic potential), M4 (high metastatic potential) and MDA-MB-231 (very high metastatic potential), begin extravasating after 12 hpi (Figure 1C). Interestingly, dissemination and extravasation patterns were also observed from injection of 15-μm fluorescent polystyrene microspheres (Figure 1D). Both, cells and polystyrene microspheres, lodge at the end of the circulatory loop (the point where the single dorsal aorta continues into the tail and then turns 180° at its most caudal end to empty into the caudal vein). These data suggest that this process is independent of the tumourigenic property of the implanted cells. After 24 h, we observed disappearance of the fluorescent signals from the transplanted cells, indicating regression of extravasated cells without initiating proliferation. Importantly, cells with moderate or high metastatic potential were capable of invading into the neighboring tail fin within 1 dpi (invasion defined as 3 to 30 cells outside of the vasculature) and subsequently develop micrometastasis (30+ cells) in the tail fin (Figure 1C). M1 cells and also the fluorescent polystyrene microspheres never showed invasion into the avascular tail fin area.

The phenomenon of mammalian cell motility is important in the progression of cancer. Interestingly, M2 and M4 cells displayed a different invasive and metastatic profile than that seen with MDA-MB-231 cells. MDA-MB-231 cell invasion is visible as singular cells into the collagen matrix of the caudal tail, whereas M2 and M4 cell invasion is visible as a collective group within the caudal haematopoietic tissue (CHT) (Figure 1E). To explore the specificity of the events we observe with the invasive breast cancer cells, we analysed the behaviour of non-tumourigenic 3 T3 mouse embryonic fibroblasts and weakly tumourigenic 293 T human embryonic kidney cells within the zebrafish embryonic model. Upon transplantation into the DoC of 2-day-old zebrafish embryos, as above, neither the 3 T3 (n = 187), nor the 293 T (n = 162) cell lines showed invasion at 6 dpi (Figure 1F). MDA-MB-231 (n = 174) cells, transplanted as a positive control, showed invasion after 12 h, and 54% of fish displayed invasion at 6 dpi. The negative control, 293 T (n = 162), did not display invasion or micrometastasis. These data imply that the process of cell invasion and metastasis within a zebrafish model occurs in a specific manner and is not merely an artefact, nor is it a passive process. Importantly, the behaviour of relatively benign M1, to pre-malignant M2 and metastatic M4 and MDA-MB-231 cells as determined in mouse and spheroid invasion assays [18,19,24] correlates with the relative state of aggressiveness of these four cell lines in the zebrafish embryo xenograft assay.

### Zebrafish and human TGF-β act cross species

Previously, we and others have shown that TGF-β has a critical role in driving invasion of M2 and M4 cells [18,25] and metastasis of MDA-MB-231 cells to bone [26-28]. Considering that the zebrafish outperforms existing mouse models with respect to high-resolution imaging of the dynamic process of cell invasion during cancer progression, and is easily amenable to genetic and pharmacological screening, we therefore set out to investigate whether the xenograft breast cancer assay in zebrafish embryos can be used to determine the role of TGF-β signalling components in breast cancer invasion and metastasis.

Our assay system is heterologous. However, as zTGF-β1 is highly homologous to higher vertebrates (Additional file 1: Figure S1A) [29], the ligands may act cross species; host-derived zTGF-β may signal on human tumour cells, and tumour-cell-derived TGF-β may bind and activate TGF-β receptors on host zebrafish cells (Additional file 1: Figure S1B-D). The zTGF-β1 construct was capable of inducing TGF-β Smad3/Smad4 mediated luciferase output significantly in two human cell lines (*P* = 0.05) (Additional file 1: Figure S1 B and C). This indicates that the zTGF-β1 interacts with the human TGF-β receptor signalling pathway. Moreover, human TGF-β is capable of inducing phosphorylated Smad2 (pSmad2), and subsequent nuclear localisation of pSmad2, in zebrafish cell lines, ZF4 and PAC2 (Additional file 1: Figure S1D). Together, these data indicate that upon injection of human tumour cells into zebrafish embryos, the two heterologous cell types may mutually communicate with each other via TGF-β receptor signalling. Furthermore, as breast cancer tumour cells, such as MCF10A and MDA-MB-231, express TGF-β themselves [18,30], we cannot exclude that human tumour cells may also mediate responses in the zebrafish embryos in an autocrine manner.

### Small molecular TGF-β type I receptor kinase inhibitors mitigate invasion of human breast cancer cells in the zebrafish model

**Figure 2:**
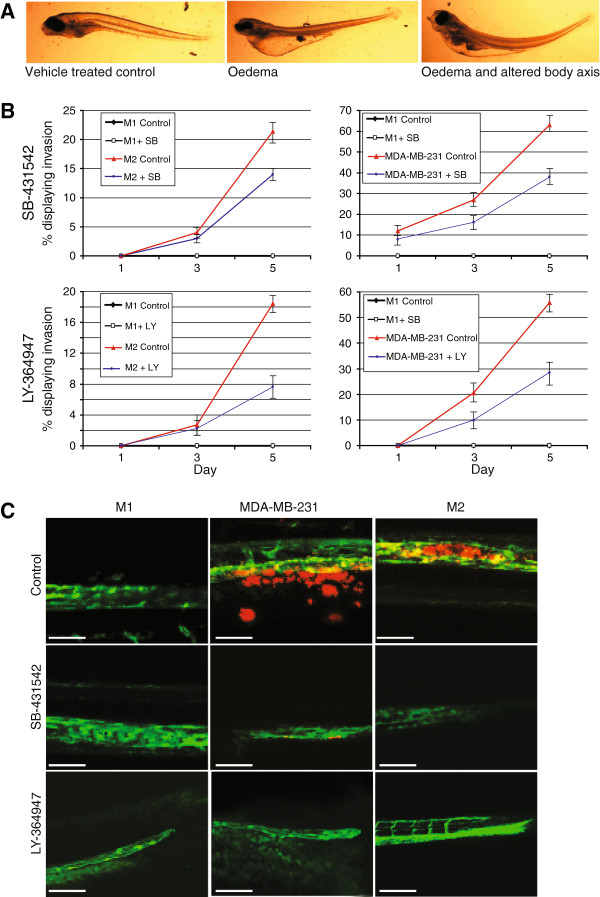
**Small molecular TGF-β receptor kinase inhibitor (TKRI) inhibited invasion of human breast cancer cells. (A)** Typical deformities seen with higher doses of SB-431542 and LY-364947. **(B)** Graphical representation of the percentage of embryonic zebrafish with invading breast cancer cells. Each group contained the non-invasive M1 cells as a control. The TKRIs were added directly to the fish water every second day. MDA-MB-231 and M2 cells are shown. Errors bars represent standard deviation. **(C)** Representative images taken at 5 days post injection. MDA-MB-231 cells have left the vasculature and are located within the collagen fibres of the tail fin. M2 cells have moved out of the vasculature and into the caudal haematopoietic tissue. All data are representative of three independent experiments with at least 50 embryos per group. Scale bar = 50 μm.

TGF-β type I receptor kinase inhibitors have been developed that selectively inhibit TGF-β responses [5], including the induction of breast cancer cell migration, epithelial to mesenchymal transition, and invasion and metastasis to bone [31,32]. Zebrafish have been used extensively for chemical screens in experimental biology [33]. Small molecules can be added directly to the water and diffuse into the zebrafish embryo [34]. We therefore set out to investigate the effect of addition of TGF-β type I kinase inhibitors on breast cancer invasion and metastasis in zebrafish xenograft assay. First we subjected the zebrafish to different doses of SB-431542 [31,32] or LY-294002 [35] TGF-β receptor kinase inhibitor (TRKI). Each inhibitor was added to the zebrafish embryos 48 hpf, and monitored for 5 days. At the doses to 5 μM or 2.5 μM of SB-431542 or LY-294002, respectively, no deformities or very few deformities were observed. Extensive malformation and death of the embryos occurred at higher doses. The typical malformations seen from intolerable doses of small molecular TRKIs included pericardial and yolk-sac oedema, altered architecture of the dorsal and caudal fins, and also short anterior-posterior body axis (Figure 2A). These effects may be (in part) on target, as deletion of TGF-β receptors also cause yolk-sac defects and pericardial effusion [36,37].

Human breast cancer cell lines, MDA-MB-231 and the MCF-10 series (M1, M2, and M4) were treated with or without the inhibitors for 24 h prior to transplantation into the zebrafish embryo (SB-431542 = 5 μM, LY-294002 = 5 μM or a vehicle control). Xenografted zebrafish were treated with the doses of the small molecular inhibitors found to be tolerable (SB-431542 = 5 μM, LY-294002 = 2.5 μM, or a vehicle control for 6 dpi). The zebrafish embryo water was changed every second day. Targeting the TGF-β pathway with small molecular inhibitors proved effective and reduced both invasion of the cancer cells, and also the development of micrometastasis (Figure 2B-C and Additional file 2: Figure S2). Furthermore, MDA-MB-231 cells that were not pretreated with the small molecular TRKI, but only treated after transplantation, also showed a reduction of invasion and micrometastasis (Additional file 3: Figure S3).

### Phospho-Smad2 expression can be switched off with small molecular inhibitors targeting the TGF-β signalling pathway

**Figure 3:**
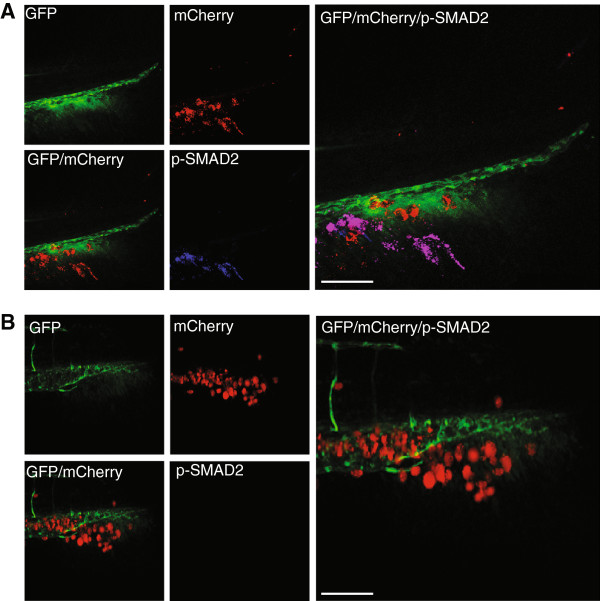
**Small molecular TGF-β type 1 receptor (TKRI) reduce expression of phosphoylated (p)Smad2.** pSmad2 expression of MDA-MB-231 cells that were not treated for 5 days post implantation (dpi), and then were treated for 1 h with **(A)** vehicle or **(B)** SB-431542. The SB-431542-treated cells were negative for TKRI activation. Data are representative of three independent experiments with at least 50 embryos per group. Scale bar = 100 μm.

Upon TGF-β type I receptor activation, Smad2 is phosphorylated at carboxy (C)-terminus on two serine residues by the TGF-β type I receptor [38,39]. C-terminal pSmad2 corresponds to activated Smad2, which is capable of forming a nuclear heteromeric complex with Smad4, which is transcriptionally active, and thus pSmad2 can be a useful tool to interrogate the activation state of the TGF-β/Smad signalling pathway. As such, zebrafish transplanted with MDA-MB-231 or M4 cells, treated with and without SB-431542 or LY-294002 (as above) were subjected to immunohistochemistry to determine if pSmad2 in tumour cells had been prevented and/or inhibited upon inhibitor treatment. Zebrafish that displayed invasion, regardless of treatment, were positive for pSmad2 (Figure 3A). This posed a conundrum, as it may be expected that invasive or metastatic cells that have overcome the inhibition of TGF-β would continue to phosphorylate Smad2, but cells that were sensitive to the treatment would not invade, and thus are not able to be examined for pSmad2. Therefore, zebrafish transplanted with MDA-MB-231 or M4 cells were kept without treatment for 5 days, and then treated with SB-431542, LY-294002 or a vehicle control for up to 24 h. This system allowed the cells to invade and metastasise, giving us the best option to visualise the impact small molecular inhibitors of the TGF-β pathway have on the zebrafish xenograft model. As seen in Figure 3B, phosphorylation of Smad2 can be effectively switched off in breast cancer cells, MDA-MB-231, when treated with a TKRI, for as little as 1 h. Thus, pharmacological inhibition of TKRI activity inhibits TGF-β/Smad2 signalling of transplanted breast tumour cells in zebrafish and inhibits their invasiveness.

### Smad4 knockdown in breast cancer cells inhibits their invasion and metastasis in the zebrafish model

**Figure 4:**
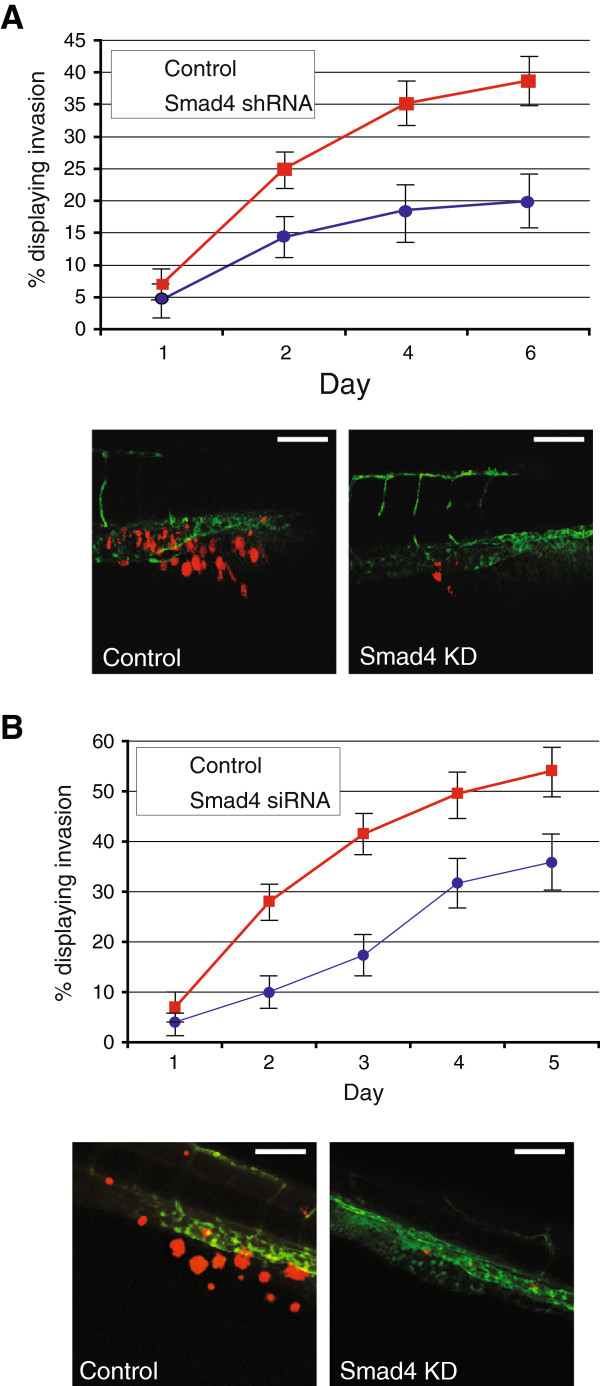
**Smad4 knockdown by shRNA or siRNA in breast cancer cells. (A)** MDA-MB-231 with stable knockdown of Smad4 displays a reduced amount of invasion. A representative image from day 6, showing cells located in the collagen fibres of the tail is shown. **(B)** Transient knockdown of Smad4 in MDA-MB-231 cells was also capable of inhibiting the amount of invasion over 5 days. A representative image from day 5 is shown. Scale bar = 100 μm. Data are representative of three independent experiments with at least 50 embryos per group. Error bars represent standard deviation.

Next we set out to examine the effect on breast cancer invasion and metastasis by antagonizing TGF-β/Smad signalling in breast cancer cells in a cell-autonomous manner. MDA-MB-231 cells with stable transfection of shRNA targeting Smad4 have been previously shown to inhibit the frequency of bone metastasis in nude mice by 75% and significantly increased metastasis-free survival in intracardiac mouse models [27]. Using the zebrafish embryo xenograft model, MDA-MB-231 cells stably transfected with shRNA targeting Smad4, or the empty vector, were examined for invasion. Similar to the mouse model, at 6 dpi, Smad4 knockdown provided a significant reduction of invasion (n = 179) (Figure 4A). Furthermore, given the relatively short time required to visualise invasion and metastasis, transient transfection of siRNA may be a useful tool. To examine the effect of siRNAs targeting Smad4 on invasion, MDA-MB-231, M2, and M4 cells were transiently transfected with Smad4-specific siRNA, or a scrambled control. Significant inhibition of invasion was seen in each cell line (Figure 4B and data not shown).

### MMP inhibition

MMPs are known to have tumour-promoting and tumour-inhibitory effects but unfortunately, several clinical trials of broad-spectrum MMP inhibitors have failed to show promising effects [40]. The specific MMP2/9 Inhibitor II, has been shown to mitigate TGF-β induced invasion of breast cancer cells [18]. Human breast cancer cell lines, MDA-MB-231 and the MCF-10 series (M1, M2, and M4) were treated with or without the 100-nM MMP2/9 Inhibitor II for 24 h prior to transplantation into the zebrafish embryo. The xenografted zebrafish were treated with 50 nM of MMP2/9 Inhibitor II, a dose of the small molecular inhibitor found to cause minimal malformation and death, or a vehicle control for 6 dpi. The zebrafish embryo water was changed every second day. Inhibiting MMP2 and MMP9 halted the invasion of metastatic breast cancer cells in the embryonic zebrafish (Additional file 4: Figure S4).

**Figure 5:**
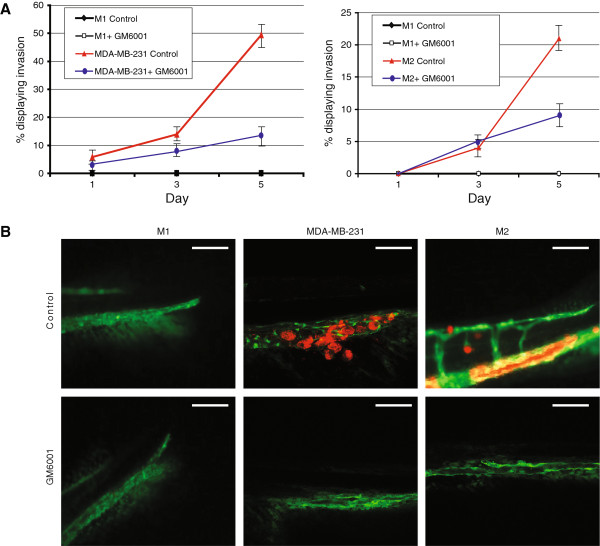
**Small molecular inhibition of matrix metalloproteinases (MMPs). (A)** Graphical representation of M2 and MDA-MB-231 cells treated with the MMP inhibitor GM6001 over 5 days. Inhibition of MMP expression reduces the amount of invasion of breast cancer cells. **(B)** Representative images of MDA-MB-231 cells that have migrated to the collagen fibres or M2 cells that have located to the caudal hematopoietic tissue. Scale bar = 100 μm. Data are representative of three independent experiments with at least 50 embryos per group. Errors bars represent standard deviation.

GM6001 is an MMP inhibitor; also known as Galardin or Ilomastat, it has been shown to suppress invasion of MDA-MB-231 cells in a three-dimensional collagen spheroid assay [41], and MCF10A M4 cells [18]. MDA-MB-231 and the MCF-10 series (M1, M2, and M4) were treated with or without the inhibitors for 24 h prior to transplantation into the zebrafish embryo (GM6001 = 10 μM). Xenografted zebrafish were treated with the doses of the small molecular inhibitors found to be tolerable (GM6001 = 5 μM), or a vehicle control for 6 dpi. The zebrafish embryo water was changed every second day. Targeting the TGF-β pathway with small molecular inhibitors proved effective and reduced the invasion of the cancer cells (Figure 5 and Additional file 5: Figure S5).

## Discussion

**Figure 6:**
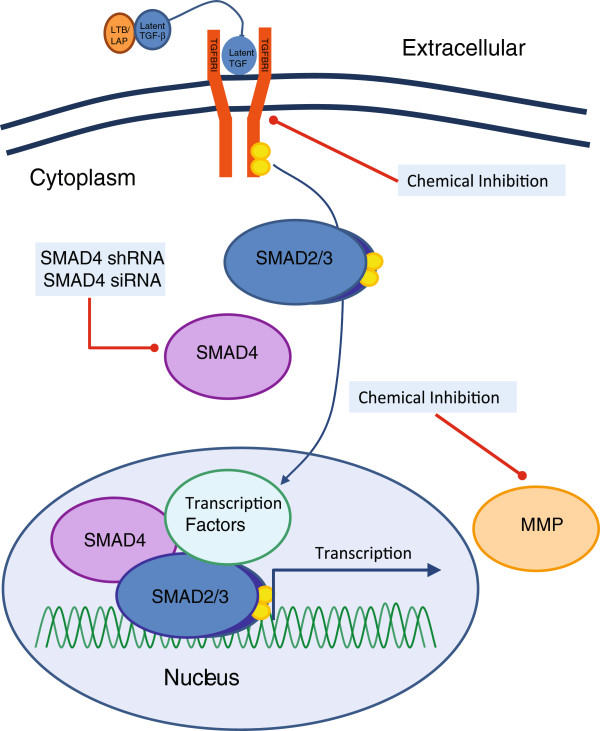
**Blocking the transforming growth factor β (TGF-β) signalling pathway.** Blocking various aspects of the TGF-β signalling pathway is capable of reducing the invasive properties of breast cells *in vivo*. The embryonic zebrafish model provides an ideal system for using chemical inhibition, siRNA, or shRNA, to target the TGF-β signalling pathway. MMP, matrix metalloproteinase.

This study used the zebrafish xenograft assay by injecting malignant breast cancer cells into the embryonic circulation, and monitoring their invasion into the avascular collagenous tail fin, as a robust and dependable animal model for examining the role of pharmacological modulators and genetic perturbation of TGF-β signalling in human tumour cells. Upon injection into the DoC, we observed that breast cancer cells dispersed immediately throughout the embryo via the circulating blood system, extravasated and invaded neighbouring tissue at specific tail-fin sites and formed micrometastasis within 6 days (as previously described in [17]). We have shown that blocking the TGF-β pathway using various methods of inhibition - and at various stages of the pathway - results in a significant reduction of invasion and metastasis of breast cancer cells (Figure 6).

Invasion and micrometastasis were only observed from breast cancer cells with metastatic potential. Interestingly, we found that M2 and M4 cells formed clusters of invasive cells mainly in the CHT region, where they are capable of proliferation (data not shown). In contrast, MDA-MB-231 cells were visible as single cells that migrated much more aggressively than M2 and M4 cells into the tail fin. It will be an interesting area of future research to determine what determines the phenotypic differences of cell migration. A recent publication has shown that ectopic expression of Slug or Snail is capable of promoting invasion of single, rounded amoeboid cells *in vitro *[42]. Furthermore, when M2 cells with Snail or Slug overexpression were implanted into the embryonic zebrafish xenograft model, single-cell invasion was also seen. This would suggest that epithelial to mesenchymal transition (EMT) is a potential requirement for single-cell migration into the tail fin. The degree of EMT within the cancer cells may also effect the communication of these cells with their environment.

Importantly, invasion could be seen at the single-cell level in the zebrafish embryo. The differences between invasive phenotypes (cells that cluster tightly together as opposed to cells that do not) are not able to be detected in larger animal models. To date, the most effective system to analyse the differences between the invasive phenotypes uses *in vitro* methods [43]. It is only due to the small, transparent nature of the zebrafish that such invasion could be seen at single-cell level in an animal model. The ability to view early-stage cell invasion *in vivo* provides an opportunity to examine the cell-cell junctions of tumours, and their environment.

We found that injected MDA-MB-231 cells showed active TGF-β/Smad signalling *in vivo* as measured by pSmad2 staining. Furthermore, we found that zTGF-β may act on human tumour cells, and tumour-cell-derived TGF-β may act on the host zebrafish. The confinement of pSmad2 staining to the tumour cells, and ability to inhibit metastasis by blocking TGF-β signalling in a tumour cell autonomous manner suggests that TGF-β produced by tumour cells acts on tumour cells to mediate cell invasion and metastasis. We were surprised not to see elevated pSmad2 staining in the host tissue surrounding the tumour cells if they secrete high levels of TGF-β. If zTGF-β would have contributed to mediating the invasion and metastatic response we would have also expected elevated pSmad2 staining in the host tissue surrounding the tumour. Further studies in zebrafish may highlight the effect that the stromal environment has on the early stages of metastatic development.

The zebrafish model described here allows simultaneous monitoring of tumour cell extravasation, invasion and micrometastasis *in vivo* within one week of implantation of human cells [17]. In other zebrafish models, cells have been transplanted into the hindbrain [21], liver, yolk [44-51], or the peritoneal cavity [11,47,52] in order to determine the invasive or metastatic potential of cancers. Although these techniques have many advantages, they also have limitations. Many of these studies show cell invasion and metastasis, which may be explained, not by the invasive properties of the cell, but by the accidental injection of cells into the blood circulation of the embryo. In some cases, invasion was shown as a singular cell that was located within the dorsal aorta. For the zebrafish embryo to be considered a dependable tool for use in investigating the process of invasion and metastasis of cancer cells, we believe that injecting cells into the DoC provides the most reliable assay. In our model, micrometastasis formation only occurs following the MDA-MB-231 cell invasion into the tail fin from the posterior end of the CHT. Previous work has shown that this invasion site is determined by the physiologic migration of neutrophils, thus supporting the so-called seed-and-soil hypothesis of tumour metastasis [17]. Furthermore, by limiting the area that we look at for invasive cells, the amount of human error is reduced. Transplanting cells into the DoC, which subsequently form a metastasis, has been shown to be controlled by the tumourigenic property of disseminated cells (as shown in Figure 2), and the microenvironment [17]. However, it is important that the conditions are optimised for each cell type, including cell number, in order that the results are reliable and reproducible.

This unique animal system provides a visual window into the metastastic process in a live vertebrate animal, with unprecedented clarity. The experiments described here establish the basis for the future development of a screening methodology for drugs that inhibit invasion and metastasis of breast cancers. Furthermore, the data also shows that transient transfection of siRNAs may be used to examine the effect of invasion and metastasis (Figures 5 and 6). We have previously used the embryonic zebrafish xenograft model to study novel regulators of the TGF-β signalling pathway, TNF receptor-associated factor 4 (TRAF4) [53] and ubiquitin-specific protease 4 (USP4) [54], as well as the tumour suppressor FAF1 [55], which interacts with the FAS ligand. The zebrafish offers a promising future for functional studies of breast cancers.

Studies of the efficacy of pharmacology and toxicology in murine xenograft models normally use tumour growth, body weight loss and mortality as parameters of toxicity. These studies are cumbersome and time consuming, and drug activity against xenografts does not always correlate with its clinical activity [56]. As seen in Figure 2, the parameters of drug dosage can be quickly and easily visualized in the zebrafish. Furthermore, small molecular compounds can be added directly to the environment of the zebrafish, which can be less stressful to the both the animal, and technician, compared to the injection techniques used in rodent models.

One potential therapy using MMP inhibition was analysed in this study using the zebrafish model. MMPs have a critical role in inflammation and tumourigenesis and appear to be ideal as a drug target. Many inhibitors have been developed, and several have gone as far as clinical trials in cancer patients. Unfortunately, though these inhibitors have showed promising effects in preclinical studies, the same agents did not have such a positive outcome in cancer treatment [2,57]. This highlights the issue of complexity that the MMPs play in cancer progression. In this study, we used GM6001, a broad-spectrum MMP inhibitor. GM6001 has been previously tested in the development of zebrafish [53,54]. These studies have shown the importance of MMPs during embryonic development [54] and fin regeneration [53]. We were able to show that MMP inhibition was capable of reducing the amount of invasion and metastasis of human mammary carcinoma cells in the zebrafish. It must be noted that MMP inhibition was initiated at the early stage of cancer metastasis. It had been previously hypothesised that, as MMPs contribute to multiple stages of tumour progression, MMP inhibition is most beneficial at the early stages and the therapeutic benefit decreases as the disease progresses [58]. Accordingly, many studies have shown that disruption of the TGF-β signalling early in metastasis can substantially reduce metastasis burden and that the effect becomes less effective when lesions become well-established [59-61]. It has been argued that well-established bone lesions may become less dependent on bone destruction and TGF-β signalling and, as a consequence, become less sensitive to TGF-β inhibitors [59].

## Conclusion

The dysregulation of TGF-β is well known in human breast cancer. Thus, the TGF-β pathway is an attractive therapeutic target. With its own advantages and disadvantages, the zebrafish embryo xenograft model represents a novel tool for investigating the tumour invasion/metastasis process. Furthermore, the zebrafish embryo xenograft model is exploitable for drug discovery and gene targeting.

## Abbreviations

CHT: Caudal haematopoietic tissue; DMEM: Dulbecco’s modified Eagle's medium; DoC: Duct of cuvier; dpf: Days post fertilisation; EMT: Epithelial mesenchymal transition; FAF1: FAS-associated factor 1; FAS: Fas (TNF receptor superfamily, member 6); FCS: Fetal calf serum; GFP: Green fluorescent protein; hpf: Hours post fertilisation; hpi: Hours post implantation; M1: MCF10A; M2: MCF10AT1k.cl2; M4: MCF10CA1a.cl1; MMP: Matrix metalloprotease; PBS: Phosphate-buffered saline; PFA: Paraformaldehyde; pSmad2: Phosphorylated Smad2; Tg: Transgenic; TGF-β: Transforming growth factor β; TRAF4: TNF receptor-associated factor 4 (TRAF4); TRKI: TGF-β receptor kinase inhibitor; USP4: Ubiquitin-specific protease 4; zTGF-β: Zebrafish transforming growth factor β.

## Competing interests

There are no competing interests to declare.

## Authors’ contributions

All authors read and approved the final manuscript. YD, SH, LZ, BESJ, and PtD designed the study. YD and SH carried out the zebrafish implantation data. YD and LZ carried out the luciferase data. YD carried out the immunohistochemical stainings, and wrote/revised the manuscript. All authors read and approved the final manuscript.

## Supplementary Material

Additional file 1: Figure S1 z-Transforming growth factor-β (TGF-β) is highly homologous to higher vertebrates. **(A)** A sequence analysis of the mature peptide from the human and zebrafish is compared. **(B)** Zebrafish (z)-TGF-βR transfected into 293 T cells was capable of inducing transcription, as indicated by the CAGA luciferase assay. **(C)** A similar result was also seen with MDA-MB-231 cells; *P* = 0.05. **(D)** Human TGF-β 1 was capable of activating the TGF-β signalling pathway in the zebrafish cell lines ZF4 and PAC2. Errors bars represent standard deviation. Click here for file

Additional file 2: Figure S2 **(A)** Graphical representation of the percentage of embryonic zebrafish with invading M4 breast cancer cells. The non-invasive M1 cells were used as a control. Error bars represent standard deviation. **(B)** Representative images taken at 5 days post injection. Scale bar = 50 μm. Click here for file

Additional file 3: Figure S3 SB-431542 acts as a strong TGF-β receptor kinase inhibitor (TKRI). **(A)** MDA-MB-231 cells, pretreated with 5 μM SB-431542 for 24 h were transplanted into 48-hours post fertilization (hpf) zebrafish embryos. No subsequent treatment of SB-431542 was given. The level of invasion was measured over 5 days. **(B)** MDA-MB-231 cells were transplanted into the 48-hpf zebrafish embryos, and treated with 5 μM SB-431542 every second day. The level of invasion was measured over 5 days. Error bars represent standard deviation. Click here for file

Additional file 4: Figure S4 Small molecular inhibition of matrix metalloproteinase (MMP)2 and MMP9. Graphical representation of M2, M4 and MDA-MB-231 cells treated with the MMP2/9 Inhibitor II over 5 days. Inhibition of MMP expression reduces the amount of invasion of breast cancer cells. Error bars represent standard deviation. Click here for file

Additional file 5: Figure S5 Small molecular inhibition of matrix metalloproteinase (MMP) with GM6001. Graphical representation of M4 cells treated with the general MMP inhibitor over 5 days. Inhibition of MMP expression reduces the amount of invasion of breast cancer cells. A representative image is included. Scale bar = 100 μm. Error bars represent standard deviation. Click here for file
